# Case Report: A rapidly growing cyst on the scalp

**DOI:** 10.12688/f1000research.19157.1

**Published:** 2019-06-04

**Authors:** Emma Short, Aisling O'Shea, Krishna Mukkanna, Girish Patel, Stefan Docjinov, Kenneth May

**Affiliations:** 1 Department of Cellular Pathology, University Hospital of Wales, Cardiff, CF14 4XW, UK; 2 Department of Dermatology, University Hospital of Wales, Cardiff, CF14 4XW, UK

**Keywords:** Trichilemmal carcinoma, pilar cyst

## Abstract

Trichilemmal carcinoma is a rare tumour derived from the outer root sheath of hair follicles ^. ^ It can be difficult to distinguish both clinically and histologically from other skin lesions, particularly squamous cell carcinoma.  We present the case of a 62-year-old female with a 20-year history of three 1-cm cysts on her scalp.  Over a six-month period, a cyst overlying the occiput had become painful and grown in size.  The general practitioner and subsequently local emergency department suspected infection.  The lesion was incised, and the patient was treated with oral antibiotics.  At the time of surgical excision, the lesion measured 3 x 4 cm.

Microscopic examination identified rounded dermal lobules of squamous epithelium with trichilemmal keratinization, in keeping with a pre-existing pilar cyst.  There were areas with nuclear pleomorphism, mitoses and an infiltrative architecture.  A diagnosis of trichilemmal carcinoma arising in a pilar cyst was made.  Trichilemmal carcinomas are considered to be a low-grade tumour, but they have the potential to spread to lymph nodes and to metastasise to distant sites in the body, therefore adequate excision and appropriate follow-up are required.

## Introduction

Trichilemmal carcinoma is a rare tumour derived from the outer root sheath of hair follicles ^1^. It typically occurs in elderly patients on sun-exposed areas of the body ^1^. Such tumours may occur *de novo*, but more commonly they arise from trichilemmal cysts, which are benign lesions arising from the isthmus of hair follicles, or proliferating trichilemmal tumours ^2^. It is thought that trauma and inflammation can induce the transformation of a benign tumour into a malignant tumour ^2^. The tumour may have a prolonged benign period before cancer develops.

This case report is important as it illustrates that a diagnosis of trichilemmal carcinoma is often delayed due to it mimicking other skin lesions.

## Case report

**Figure 1.**
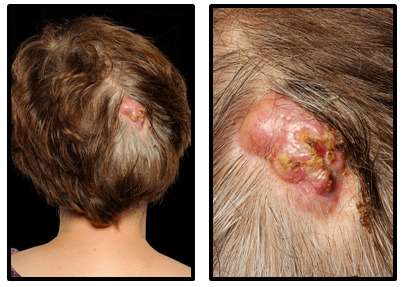
Clinical examined identified a 3 x 4 cm raised, indurated lesion with crusting, superficial ulceration and a serosanguinous discharge.

A 62-year-old Caucasian British female presented with a 20-year history of three 1-cm cysts on her scalp. She was previously fit and well and had no significant medical history. Over a seven-month period, the cyst overlying the occiput had become painful and grown in size. During this time, the patient had visited her general practitioner and local emergency department, both of which suspected infection. The lesion was incised, and the patient was treated with three courses of oral flucloxacillin (each course, 500 mg four times per day for 1 week) and one course of oral clarithromycin (250 mg twice per day for 1 week). At the time of surgical excision, the lesion measured 3 x 4 cm. It was raised, indurated, crusted, demonstrated a sparsity of hairs on the surface, had superficial ulceration and exuded serosanguinous fluid when pressed ( Figure 1). There was no palpable lymphadenopathy.

**Figure 2.**
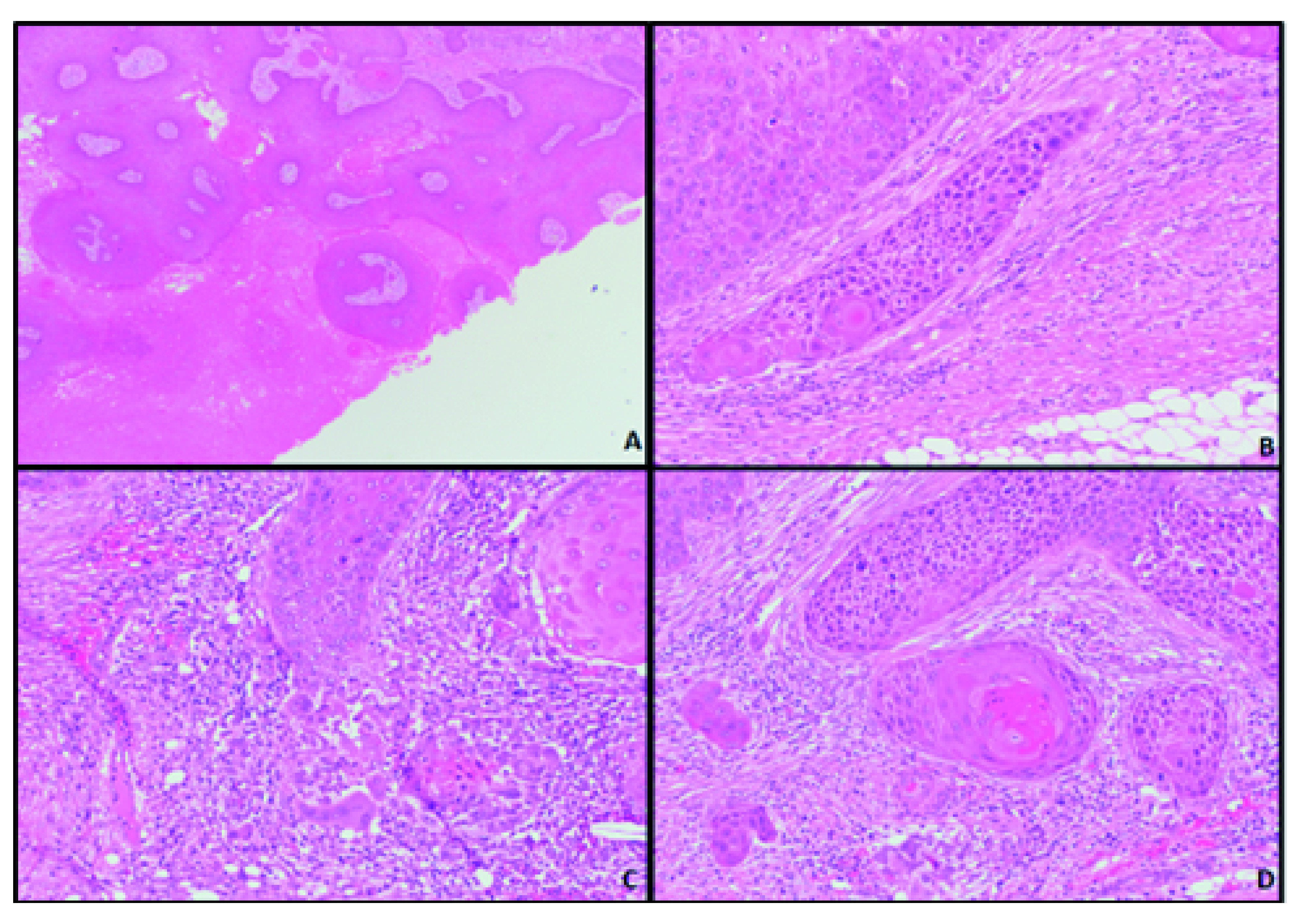
Microscopic images of the skin tumour. ( **A**) Squamous epithelium with trichilemmal keratinisation (x4 objective). ( **B**– **D**) Epithelium with nuclear pleomorphism, mitoses and an infiltrative architecture (x20 objective).

Microscopic examination of the lesion identified rounded dermal lobules of squamous epithelium with trichilemmal keratinisation in keeping with a pre-existing pilar cyst ( Figure 2A). Areas with nuclear pleomorphism, mitoses and an infiltrative architecture were noted, and they retained trichilemmal keratinisation ( Figure 2B–D). The features were of a trichilemmal carcinoma arising in a pilar cyst.

She was reviewed three months post-operatively. The wound had healed well and there was no sign of recurrence.

## Discussion

Trichilemmal carcinomas can be difficult to distinguish clinically and histologically from other skin lesions, particularly squamous cell carcinoma (SCC). Microscopically they are characterised by an abrupt transition of nucleated squamous epithelial cells to keratinised cells, without the formation of a granular layer ^3^, and a lobular proliferation of epithelial cells which exhibit nuclear pleomorphism, prominent mitotic activity and infiltration beyond the basement membrane ^4^.

Trichilemmal carcinomas are considered to be a low-grade tumour, but they do have the potential to spread to lymph nodes and to metastasise to distant sites in the body ^5^. There are also reports of death due to the disease ^6^. Therefore, prompt treatment is necessary to reduce morbidity and mortality. Surgical excision with a 1-cm border is the recommended treatment. However, in recent years, Mohs surgery has been used with success.

For recurrent disease, or cases with lymph node or distant metastases, radiotherapy and chemotherapy are sometimes considered, but often there is no standard protocol for trichilemmal carcinoma treatment, and regimens similar to those used for SCC are employed. Following treatment, patients will need to undergo regular follow-up due to the risk of recurrence and/or metastases. Because of the tumour’s rarity, standard treatment and follow up protocols have not been established.

## Conclusion

Trichilemmal carcinoma is a rare adnexal tumour. It can mimic common skin lesion such as cysts or squamous cell carcinoma. Diagnosis is dependent on microscopic examination, and the identification of features including the absence of a granular cell layer, a lobular architecture, cellular pleomorphism, mitoses and invasion beyond the basement membrane. The tumour can behave aggressively. Adequate excision and appropriate follow-up are required.

## Learning points

- Trichilemmal carcinoma is a rare adnexal tumour.
- It can mimic common skin lesion such as cysts or squamous cell carcinoma.
- Diagnosis is dependent on microscopic examination, and the identification of features including the absence of a granular cell layer, a lobular architecture, cellular pleomorphism, mitoses and invasion beyond the basement membrane.
- The tumour can behave aggressively. Adequate excision and appropriate follow-up are required.

## Data availability

All data underlying the results are available as part of the article and no additional source data are required.

## Consent

Written informed consent for publication of their clinical details and clinical images was obtained from the patient.
