# Supplementary material for: Case Report: A rapidly growing cyst on the scalp
Source: F1000Res. 2019 Jun 4;8:779. [Version 1] doi: 10.12688/f1000research.19157.1 (PMC7739100; doi:10.12688/f1000research.19157.1)
Supplement: Peer review history

# Peer review: Reviewer response for version 1

**DOI:** 10.5256/f1000research.20993.r76140

**Published:** 2020-12-15

**Authors:** Manas Dave

**Affiliations:** 1 NIHR Academic Clinical Fellow in Oral and Maxillofacial Pathology, The University of Manchester, Manchester, UK

Introduction

 Well written introduction acknowledging trichilemmoma and its malignant variation. 

 Case report

 Grammatical considerations - General Medical Practitioner should be used instead of General Practitioner

 Well documented photos and descriptions; are higher resolution histology images available? 

 Discussion

 Well written - the authors may wish to add that this case report exemplifies the importance of a comprehensive microscopic examination for seemingly clinically benign pilar cysts and the importance of a thorough histological assessment. This can also be added into the learning points. 

 Consent from the patient acknowledged

 This is a well written case report by the authors presenting a uncommon malignancy.

Are enough details provided of any physical examination and diagnostic tests, treatment given and outcomes?

Yes

Is the case presented with sufficient detail to be useful for other practitioners?

Yes

Is sufficient discussion included of the importance of the findings and their relevance to future understanding of disease processes, diagnosis or treatment?

Yes

Is the background of the case’s history and progression described in sufficient detail?

Yes

Reviewer Expertise:

Histopathology

I confirm that I have read this submission and believe that I have an appropriate level of expertise to confirm that it is of an acceptable scientific standard.

# Peer review: Reviewer response for version 1

**DOI:** 10.5256/f1000research.20993.r64756

**Published:** 2020-06-29

**Authors:** Toshitsugu Nakamura

**Affiliations:** 1 Department of Diagnostic Pathology, Ina Central Hospital, Ina, Japan

This article describes a rare case of trichilemmal carcinoma (TC).  It may be valuable, from educational aspect, for the practitioners who have not encountered such a rare tumor, with a condition that pathological diagnosis is accurate and strict. It seems to me, however, that Figure 2 does not show characteristic features for TC or pilar cyst (PC). Although the microphotographs in Figure 2 are somewhat out of focus and are not discernible enough, the tumor looks squamous cell carcinoma (SCC). I suggest the authors to present characteristic histopathological features for TC/PC, as follows:

 Figure 2A:

1. Low-power view showing cystic structure
2. Distinct feature of trichilemmal keratinization.

Figures 2B/2C/2D:

1.  Low-power view showing folliculo-centric structure of the tumor
2. Distinct feature of trichilemmal keratinization (Absence of granular cell layer is not a warrant of trichilemmal keratinization, because conventional SCC usually shows keratinization from squamoid tumor cells without interposition of granular cell layer)
3. The tumor cells with clear cytoplasm, stained by periodic acid-Schiff (PAS) procedure.

In case that the present tumor is difficult to distinguish from SCC, immunohistochemical markers for trichilemmal differentiation, such as CD34 and CK17, should be examined and presented.

Are enough details provided of any physical examination and diagnostic tests, treatment given and outcomes?

Yes

Is the case presented with sufficient detail to be useful for other practitioners?

No

Is sufficient discussion included of the importance of the findings and their relevance to future understanding of disease processes, diagnosis or treatment?

Partly

Is the background of the case’s history and progression described in sufficient detail?

Yes

Reviewer Expertise:

diagnostic pathology, immunohistochemistry, pathology of cell death and terminal differentiation

I confirm that I have read this submission and believe that I have an appropriate level of expertise to confirm that it is of an acceptable scientific standard, however I have significant reservations, as outlined above.

## References

[1] WHO Classification of Skin Tumours. *IARC Publications*.2018;199-200

# Peer review: Reviewer response for version 1

**DOI:** 10.5256/f1000research.20993.r49455

**Published:** 2019-07-02

**Authors:** Laszlo Igali

**Affiliations:** 1 Norfolk and Norwich University Hospital (NNUH), Norwich, UK; 2 University of East Anglia, Norwich, UK

This article highlights the difficulties in the clinical and histological diagnosis of a rare adnexal tumour. The number of properly described bona fide trichilemmal carcinomas is low, therefore case reports describing the observed histological features are  valuable. The authors also highlighted the long clinical history related to a pre-existing pilar cyst - transformation of the tumour can occur, sometimes in a long and unpredictable clinical course.

 I would suggest one area, which could be expanded more is the differential diagnosis (perhaps use and differentiating value of immunohistochemistry) as well as it's relationship to the follicular/infundibular squamous cell carcinoma.

 I would recommend indexing this article as it is - the short and succinct description and important clinical and histological points make this useful, and the point raised above is probably beyond the scope of a case report.

Are enough details provided of any physical examination and diagnostic tests, treatment given and outcomes?

Yes

Is the case presented with sufficient detail to be useful for other practitioners?

Yes

Is sufficient discussion included of the importance of the findings and their relevance to future understanding of disease processes, diagnosis or treatment?

Yes

Is the background of the case’s history and progression described in sufficient detail?

Yes

Reviewer Expertise:

Dermatopathology, melanocytic disorders and skin cancer

I confirm that I have read this submission and believe that I have an appropriate level of expertise to confirm that it is of an acceptable scientific standard.
